# Ectopic bronchogenic cysts in uncommon abdominal locations: A case report of splenic involvement

**DOI:** 10.1097/MD.0000000000043486

**Published:** 2025-08-01

**Authors:** Qi Liu, Guanying Yu, Houjun Zhang, Peiming Guo, Chengzhen Li

**Affiliations:** a Department of Gastrointestinal Surgery, Central Hospital Affiliated to Shandong First Medical University, Jinan, China; b School of Clinical Medicine, Shandong Second Medical University, Weifang, Shandong, China.

**Keywords:** ectopic bronchogenic cyst, misdiagnosis prevention, splenic involvement, surgical management

## Abstract

**Rationale:**

Ectopic bronchogenic cysts (EBCs) are rare congenital lesions, mostly located in the mediastinum. They are extremely rare in the abdomen, particularly near the spleen.

**Patient concerns:**

A 48-year-old Chinese female, who was asymptomatic and undergoing a routine health examination, was incidentally found to have a low-density soft tissue mass near the spleen. Preoperative considerations included a mucinous tumor, a gastrointestinal stromal tumor, or malignant tumors such as gastric cancer.

**Diagnoses:**

A contrast-enhanced computed tomography scan showed a 2.6 cm soft tissue mass between the stomach and spleen. Gastroscopy revealed no significant abnormalities, and laboratory tests were normal. Laparoscopic exploration identified the mass in the splenic fossa, and histopathological examination confirmed it as an ectopic bronchogenic cyst.

**Interventions:**

The patient underwent laparoscopic resection of the mass, involving dissection of adhesions and separation from the spleen.

**Outcomes:**

The patient recovered uneventfully postoperatively and was discharged after six days. Follow-up computed tomography at four months showed no recurrence, and the patient remained asymptomatic.

**Lessons:**

Recognizing ectopic bronchogenic cysts in rare abdominal locations, like the spleen, is essential for proper diagnosis and treatment. Early intervention for suspected malignant tumors, especially with minimally invasive laparoscopic surgery, is key to effective management and can make a significant difference in patient outcomes.

## 1. Introduction

Bronchogenic cysts (BCs) are rare congenital lesions, most commonly found in the mediastinum, but can also occur in ectopic locations. Ectopic bronchogenic cysts (EBCs) in the abdominal region are extremely rare, particularly near the spleen. The incidence of BCs is approximately 1 in 68,000 to 1 in 42,000, with mediastinal cysts accounting for 90% of cases.^[1,2]^ EBCs are believed to arise from abnormal development of bronchial buds during the 3rd to 6th weeks of embryonic development. These displaced bronchial buds may migrate to various sites, leading to ectopic cyst formation.^[3]^ Clinically, EBCs are often asymptomatic, especially in their early stages. As they enlarge, they may cause symptoms such as abdominal pain, bloating, and belching. If the cyst becomes infected, fever and pain may occur, and in some cases, gastrointestinal bleeding may follow.^[4,5]^ Malignant transformation of BCs is rare but has been reported.^[6]^ Imaging studies, including computed tomography (CT) and MRI, are helpful for detecting EBCs. These cysts typically appear as low-density, well-defined lesions with little or no enhancement on imaging. While imaging can help identify the cyst’s size, location, and relationship to surrounding organs, definitive diagnosis often requires surgical resection and histopathological examination.^[7]^ This case report describes an unusually located EBC near the spleen, emphasizing the diagnostic challenges and the complexity of managing such a rare condition in the abdomen.

## 2. Case report

**Figure 1.**
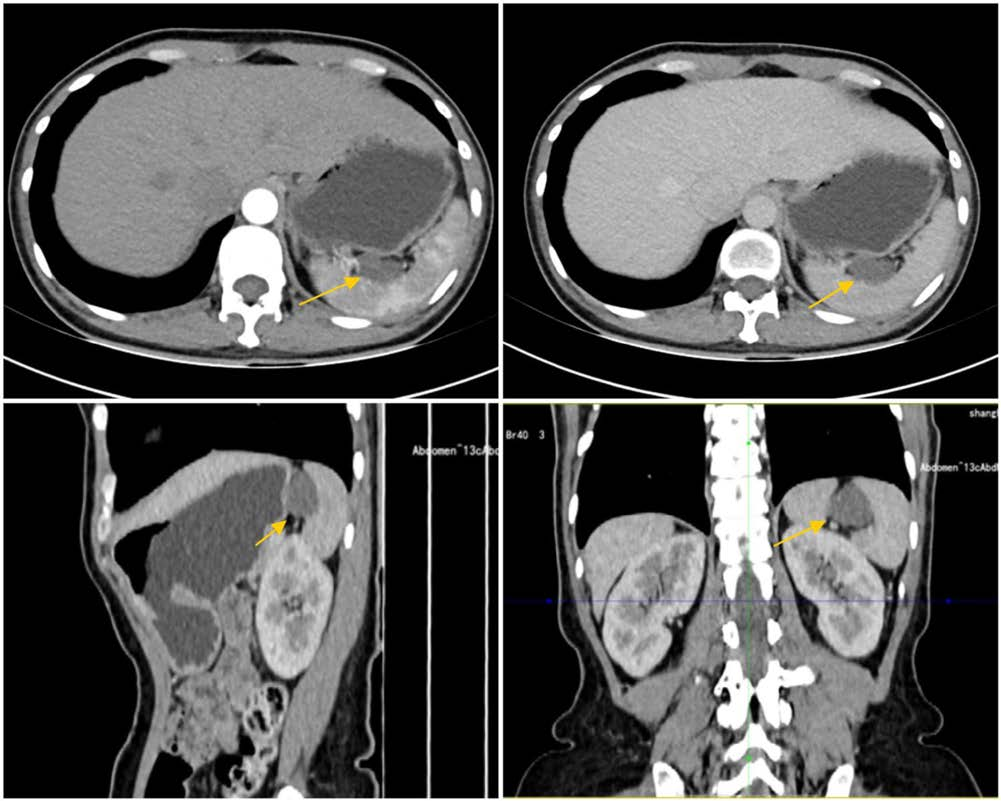
Contrast-enhanced CT images demonstrating the relationship between the tumor and the spleen in axial, sagittal, and coronal planes. CT = computed tomography.

A 48-year-old Chinese female patient was admitted for a routine health examination. She reported no symptoms, including weight changes or discomfort, and had no significant medical history, including no known chronic diseases or family history of malignancy. Physical examination revealed no palpable masses or other abnormal findings. A contrast-enhanced CT scan of the chest and abdomen was performed, revealing a low-density soft tissue mass between the stomach and spleen, with a maximum diameter of 2.6 cm. A contrast-enhanced CT scan of the chest and abdomen revealed a low-density soft tissue mass at the edge of the spleen, with a maximum diameter of 2.6 cm. The mass showed no significant enhancement during the arterial phase and appeared avascular, closely associated with the posterior wall of the gastric fundus and the spleen, with indistinct borders (Fig. 1). The initial diagnosis suspected a mucinous tumor of the spleen.

**Figure 2.**
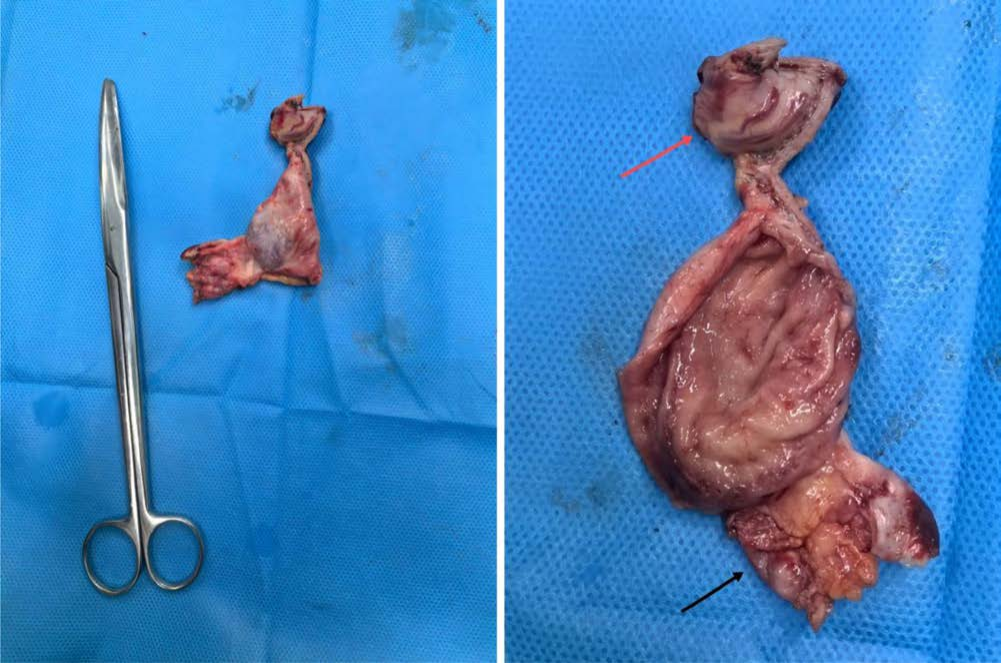
The opened mass. Partial gastric wall (red arrow), splenic upper pole (black arrow).

Subsequent gastroscopy revealed a gastric polyp with no evidence of mucosal disruption or external compression. Laboratory tests, including tumor markers, were within normal limits. Given the ambiguous nature of the lesion, laparoscopic exploration was performed, revealing no metastasis or ascites. The mass, measuring approximately 2.5 cm, was found in the splenic fossa (Fig. 2). It was soft with poorly defined borders and appeared to infiltrate the upper pole vessels of the spleen. The ultrasonic scalpel was used to carefully dissect the gastrocolic ligament and separate the mass from the spleen, a process complicated by dense adhesions. During dissection, the mass ruptured, releasing a mucinous substance. There was bleeding from the upper pole vessels of the spleen, which was controlled with absorbable titanium clips. The procedure concluded without complications, and no active bleeding was noted.

**Figure 3.**
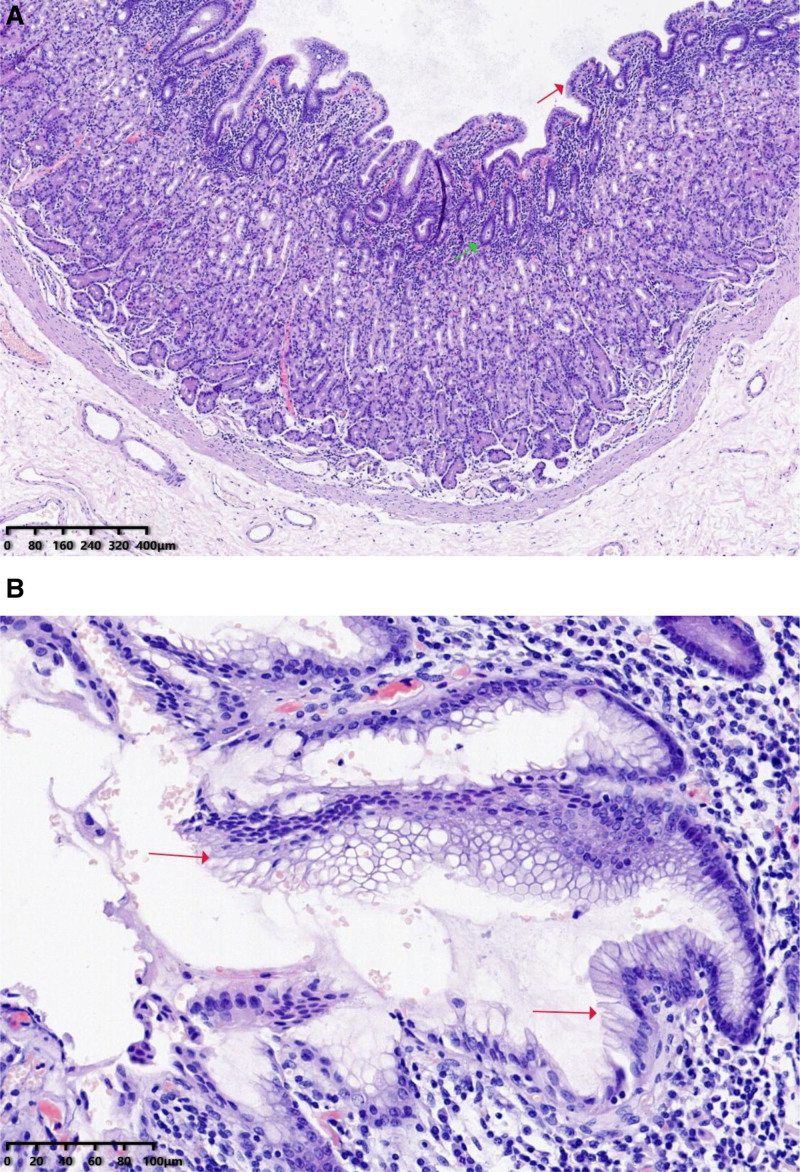
Histopathological examination with hematoxylin and eosin staining (50× and 200× magnification): The cyst is composed of smooth muscle and pseudostratified ciliated columnar epithelium (red arrow). There is no cellular atypia, and the stroma (green arrow) contains serous and mucous glands.

Histopathological examination of the excised tissue revealed a fibrous cyst wall lined by ciliated columnar epithelium and pseudostratified columnar epithelium, consistent with a bronchogenic cyst (Fig. 3). The patient recovered uneventfully and was discharged on postoperative day 6 with a small amount of serous drainage from the splenic fossa, which was expected to resolve spontaneously. At a 4-month follow-up, the patient underwent a CT scan at a local hospital, which showed no signs of recurrence. The patient remained asymptomatic.

## 3. Discussion

BCs are congenital benign lesions derived from aberrant embryologic development of the foregut, primarily characterized by bronchial-type mucinous and smooth muscle tissue.^[2]^ While most commonly located in the mediastinum, ectopic cases are rare, with occurrences in cervical, thyroid, chest wall, diaphragmatic, and retroperitoneal regions.^[2,3,8]^ Intra-abdominal BCs are exceptionally uncommon, typically presenting near the stomach.^[5]^ Splenic BCs are particularly rare, with no documented cases in the literature. In this report, we present a case of splenic EBC to enhance clinical awareness and minimize diagnostic errors.

Preoperative diagnosis of abdominal EBCs is challenging due to their high misdiagnosis rate. Abdominal EBCs typically lack specific clinical symptoms and are often incidentally discovered during routine examinations.^[9]^ In certain cases, cyst enlargement may cause compressive symptoms in adjacent organs, while secondary infections can lead to fever and pain.^[4]^ Imaging techniques remain central to EBC diagnosis. Ultrasound can reveal the size and location of masses, but often fails to identify the cyst origin. CT scans typically display well-defined, round or oval, low-density lesions with a uniform density (CT value of 0–100), showing little to no enhancement. Contrast-enhanced CT and MRI help characterize the cyst, elucidating its location, borders, morphology, and vascular relationships, which is critical for surgical planning.^[7]^ MRI findings generally include T1 and T2 hyperintense signals, with T2-weighted sequences showing high signal intensity when fat is suppressed.^[10]^ However, definitive diagnosis requires surgical resection and histopathological examination to differentiate from gastrointestinal stromal tumors and splenic mucinous neoplasms.

Surgical resection remains the primary treatment for bronchogenic cysts, despite ongoing debates regarding the timing of surgery. Although malignant transformation is rare,^[11]^ potential complications include ulceration, infection, and hemorrhage.^[12]^ In the absence of contraindications, we recommend complete surgical resection of the cyst wall to prevent recurrence. Most patients experience favorable postoperative recovery with no recurrence or metastasis observed during follow-up.

In conclusion, recognizing ectopic bronchogenic cysts in uncommon abdominal sites, as exemplified by splenic involvement, is essential for broadening differential diagnoses and informing precise surgical interventions to prevent misdiagnosis.

## Author contributions

**Writing – original draft:** Qi Liu.

**Writing – review & editing:** Guanying Yu, Houjun Zhang, Peiming Guo.

**Conceptualization:** Chengzhen Li.

**Funding acquisition:** Chengzhen Li.

**Resources:** Chengzhen Li.
